# Evaluation of spiritual well-being in women with breast cancer

**DOI:** 10.1192/j.eurpsy.2026.11559

**Published:** 2026-07-31

**Authors:** N. Bouattour, S. Omri, W. Ben kridiss, N. Charfi, N. Smaoui, I. Gassara, R. Feki, M. Mâalej, A. Khanfir, M. Mâalej, L. Zouari

**Affiliations:** 1 Psychiatry C, Hedi Chaker University Hospital; 2 Medical Carcinology, Habib Bourguiba University Hospital, Sfax, Tunisia

## Abstract

**Introduction:**

The process of undergoing chemotherapy is a long, tiring, and challenging phase of the treatment for a woman with breast cancer. Inner support, like spiritual well-being, plays a key role in this uneasy period.

**Objectives:**

To assess spiritual well-being in patients with breast cancer undergoing chemotherapy and to identify factors associated with better spiritual well-being.

**Methods:**

We conducted a descriptive and analytical cross-sectional study with breast cancer patients in January 2025, at the day care unit in the Medical Carcinology department at the Habib Bourguiba University Hospital. We used a preestablished sheet that contained the sociodemographic and therapeutic characteristics. We assessed spiritual well-being using the Spiritual Well-Being Scale (SWBS), which contains two dimensions: Religious Well-Being Score and Existential Well-Being Score.

**Results:**

Our study involved 47 women diagnosed with breast cancer for at least 3 months. The mean age was 51.78 ±11.62 years old. The majority of the participants were married (80.9%, n=38). For the educational level, 87.3% were educated. Socioeconomic status was low at 87.2%. Urban geographic origin was 76.6% (n=36). Six participants (12.8%) had a family history of cancer. Metastatic stage was found among 51.1% of the participants (n=24). Improvement in response to chemotherapy was shown in 68.1% (n=32) compared to 12.8% (n=6). For the remaining participants (19.1%, n=9), additional time was required to evaluate the response to the treatment.

The mean score of the SWBS was 79,76± 14,31. The mean score of the religious Well-Being was 45,59 ± 5,12, and of the existential Well-Being Score was 35,06 ± 11,58. The absence of metastases was statistically associated with a higher score of the SWBS (P<10 -3). Improvement in response to chemotherapy was statistically associated with higher spiritual well-being (P<10 -3). Participants with more than 12 months of follow-up demonstrated better scores in the SWBS scale (P=0.042).

No statistical association with the marital status, educational level, or family history of cancer was found in the study.

**Conclusions:**

Our study highlights the importance of spiritual well-being among women with breast cancer as an indicator of the overall satisfaction with their lives. This dimension can help them cope with the disease and, therefore, go through the journey of long-term medication.

**Disclosure of Interest:**

None Declared

